# Global Positioning System Analysis of Physical Demands in Small and Large-Sided Games with Floaters and Official Matches in the Process of Return to Play in High Level Soccer Players

**DOI:** 10.3390/s20226605

**Published:** 2020-11-18

**Authors:** Demetrio Lozano, Miguel Lampre, Adrián Díez, Oliver Gonzalo-Skok, Diego Jaén-Carrillo, Daniel Castillo, José Luis Arjol

**Affiliations:** 1 Health Sciences Faculty, Universidad San Jorge, Autov A23 km 299, Villanueva de Gállego, 50830 Zaragoza, Spain; miki_tauste@hotmail.com (M.L.); adri.diez.1991@gmail.com (A.D.); oligons@hotmail.com (O.G.-S.); djaen@usj.es (D.J.-C.); jlarjol@usj.es (J.L.A.); 2 Faculty of Health Sciences, Universidad Isabel I, 09003 Burgos, Spain; danicasti5@gmail.com

**Keywords:** GPS, team sport, match monitoring, physical performance, peak velocity

## Abstract

The aim of this study was twofold: (i) to analyze the physical demands in the return to play (RTP) process of high-level soccer players in the role of floater in different soccer sided games (SGs) formats (i.e., 4vs4 + 2 and 8vs8 + 1); and (ii) to analyze the differences in physical demands encountered by regular and floater players among the SGs formats and official matches by means of global positioning system technology (GPS APEX pod, North Ireland) was used. Twenty-six highly trained, male soccer players (U16 years) participated in this investigation. Players were classified into two groups: 23 regular and 3 floater players, a total of eight SGs were analyzed, which involved the recording of 80 observations of regular and floater players. Match-play players showed most likely–probable differences in distance covered at high-intensity per minute (D > 14.4/min), at high-speed running per minute (D > 21/min), and peak velocity (Vpeak) in comparison to floaters in the 8vs8 + 1 LSG (large-side-games), and presented most likely differences in accelerations >2/min in comparison to match-play players. Therefore, the use of floaters during the last phase of the RTP (return to play) seems to be a useful strategy for progressive reintroduction into specific training (1) floater in the 4vs4 + 2 SSG; (2) floater in the 8vs8 + 1 LSG; (3) regular player in the 4vs4 + 2 SSG; and (4) regular player in the 8vs8 + 1 LSG before starting full trainings and returning to competition.

## 1. Introduction

The return to play (RTP) process is defined as the phases through which an injured athlete safely returns to training and the competition context in order to protect his health, decreasing the risk of reinjury, and minimising the time when the athlete is unavailable for competition. The RTP is a continuous process comprised of three phases: return to participation, return to normal training, and return to successful performance in competition. The RTP is as functional sports re-education, differentiating three phases: recovery, retraining, and training. These phases could ensure an optimal adaptation and the progressive improvement of performance thanks to the application of specific external loads, and consequently allow players to reach a successful RTP.

Soccer sided games (SGs) have usually been used as a training methodology in soccer because they allow players to replicate specific behaviors during the development of the game and they resemble the physical, technical, and tactical responses demanded during competition [1,2]. SGs face two teams in a collaboration–opposition context involving simultaneous space and participation [3]. These games are played in limited playing areas with adapted rules and a reduced number of players [1]. Under previous literature [4], SGs have been classified based on the number of players per team as small-sided games (SSGs) using 2–4 players, medium-sided games (MSGs) involving 5–7 players, and large-sided games (LSGs) using 8–11 players. Considering these SGs formats, it would be adequate to take into account the number of players in the RTP process, since this would allow athletes to progressively and specifically prepare to face the competition in compliance with the specificity training principle [5].

During the last phase of the RTP process, it would be appropriate to monitor the physical demands of the SGs in terms of external loads. Since the distribution of the training loads is crucial to successful performance, the individual training load of players who return to play should be individualised [6]. To periodize an adequate progression of the physical demands during the RTP process [7], it would be convenient to establish a distribution focused on recovery from injuries. Thus, coaches should plan an optimal and progressive transition from individual work to teamwork, adjusting the efforts, sessions and initial tasks with the team [8]. In this training context, players must train with physical demands of progressive volume and intensity and with integration of specific tactical and technical demands in order to avoid compromising their health and a possible reinjury [8]. Thus, it would be interesting to quantify the physical demands during different SGs formats and according to playing roles (i.e., regular and floater players) during the RTP process.

The floater is a special player who belongs to the team in possession of the ball during the development of the SGs [9], allowing teams a numerical superiority and participating always in the offensive phase [10]. Lacome et al. (2018) suggested that the use of floaters could be advisable for players who need lower physical demands; that is, recently injured players. Rábano-Muñoz et al. (2019) showed that floaters registered lower external training loads in comparison to regular players with respect to peak velocity (Vpeak) and maximum heart rate [11]. In this line, Lacome et al. (2018) reported that total distance, distance at high-intensity speeds (>14.4 km·h^−1^), accelerations (>2 m·s^−2^), decelerations (<−2 m·s^−2^), and changes of direction were lower in the floaters compared to regular players independently of the SG format [9]. However, Hill-Haas et al. (2010) found that floaters covered greater total and sprint distances than regular players in SSG and LSG formats. Due to this controversy in the aforementioned studies, it seems necessary to examine the physical demands encountered by floaters and regular players in different SG formats.

Since the main risk of injury for players could be to have previously been injured [12], it would be very relevant to monitor the training loads during SGs in the RTP process in order to optimise their physical performance. Therefore, the aim of this study was twofold: (i) to analyze the physical demands in the RTP process of high-level soccer players in the role of floater in different SGs formats (i.e., 4vs4 + 2 and 8vs8 + 1); and (ii) to analyze the differences in physical demands encountered by regular and floater players among the SGs formats and official matches.

## 2. Materials and Methods

A cross-sectional, comparative study design was used. One week before the beginning of the investigation, two familiarization sessions were carried out which included both the use of global positioning system (GPS) devices and the realization of the different SG formats. The intervention protocol was performed in the middle of the season (February–March), during the same training session at least 72 h after the last official match and match-play. The SSGs were always performed at the usual training time of the team (17:00–18:30 h), with the players wearing their training uniforms and soccer boots to play on the artificial grass field on which they normally trained. During the task each group of regular players wore a different color t-shirt and floaters wore a different color than regular players. The distribution of both groups, regular players, and floater players, was random, and no player was in a process to return to play. During the duration of the study, the players and their parents were instructed to maintain their usual habits, which included eight hours of night-time sleep before each data collection session, adequate hydration, and carbohydrate intake over the 24 h before each experimental SSG [13]. Players that no played whole match or played less to 60 min did complementary training to equal the physical demands and the weekly load of the players that played whole match. During the duration of the study, the physical demands encountered by soccer players in the SSG 4vs4 + 2 floaters and in the LSG 8vs8 + 1 floater were recorded, differentiating regular and floater players. Prior to the SGs, a standardized warm-up established by the researchers and technical staff was performed, consisting of 5 min of low intensity running, 3 min of mobility exercises, and 2 min of active stretching and ballistic exercises. Finally, technical–tactical drills were played for 10 min.

### 2.1. Subjects

Twenty-six highly trained, male soccer players under the age of 16 participated in this investigation (age: 15.7 ± 0.3 years; height: 176.1 ± 5.8 cm; body mass: 68.0 ± 5.6 kg; sitting height: 91.7 ± 3.0; leg length: 84.4 ± 3.2; age at peak height velocity [APHV] 13.8 ± 0.4, calculated according to [14]). Players were classified into two groups: 23 regular and three floater players. Table 1 shows the characteristics of the participants. Soccer players were involved in their usual trainings of 4–5 sessions of 60–90 min per week and an official match during the weekend. Medical staff reviewed the medical records and assessed the players’ suitability for their participation in the investigation, and those players who were injured were excluded [15]. All players were informed about the procedures, the objectives and the experimental protocol before giving their written consent. In addition, all parents or guardians of players under 18 years of age signed the consent was obtained. The investigation was performed in accordance with the Declaration of Helsinki 1964 and was approved by a Local Ethics Committee (USJ-042021).

### 2.2. Procedures

Small-sided games and official match-play. Participants carried out two SG formats (Table 2): The SSG 4vs4 + 2 floaters and the LSG 8vs8 + 1 floater. All tasks and sets were random in their distribution. SSG and LSG were performed in two periods of 6 min, interspersed with 3 min of passive recovery. All series of each SG was analyzed, and rest periods were excluded from the analysis. Two kind of SG were included in the session four days before competition and aimed to develop the players’ strength and power capabilities (area: <100 m^2^ per player) (Martín-García et al., 2018). One week we implemented SSG task and the next week we implemented LSG task during eight weeks. A total of eight SGs were analyzed, which involved the recording of 80 observations of regular and floater players. The objective of the SGs was to maintain possession of the ball for as long as possible allowing free touches per player. We did not use goals because we did not have available goalkeepers. The coach was required to give verbal encouragement and to introduce balls immediately when the ball left the playing field [16]. Moreover, we analyzed four official matches, which involved the recording of 38 observations of football players along four weeks (One match per week) that the intervention lasted. During the match-play, only data were taken from the players who completed the entire match (80 min). Each match was composed of two halves of 40 min whit 15 min of rest. All matches were scheduled at the same time of the day (12:00 hours). The data was extracted through the specific software Apex, Statsports, Ireland, Version 1.2.

Physical demands. To quantify the physical demands during training sessions and matches, the GPS APEX pod (18Hz GPS, 600Hz accelerometer, MAPPS Technology and Bluetooth LE; Statsports; North Ireland) was used. Geometrically position dilution of precision (PDOP) was estimated at 1.290–1.880. APEX units (18Hz) showed good levels of accuracy (bias < 5%) in sport specific metrics [17]. Microsensor units were harnessed in a tight-fitting vest that was worn by the soccer players during the experimental study [18,19]. The microsensor devices were activated 15 min before the start of each testing session, in accordance with the manufacturer’s recommendations. This period of time was excluded from the study. Data were downloaded post-SSG protocol to a computer and analyzed using a customized software package (Statsports Apex, North Ireland). To minimize inter-devices error, each floater player used the same GPS during the intervention period [18,19]. Physical demands were expressed as relative measures according to the playing time (m·min^−1^) or attending to the maximum peak velocity. The physical demands recorded were: total distance covered per minute (TD/min), distance covered at low-intensity (<14.4 km·h^−1^) (D < 14.4/min), distance covered at high-intensity per minute (>14.4 km·h^−1^) (D > 14.4/min), distance covered at high-speed running per minute (>21.0 km·h^−1^) (D > 21.0/min), number of accelerations (>2 m·s^−2^) per minute (Acc > 2/min), number of decelerations (>2 m·s^−2^) per minute (Dec > 2/min), and peak velocity (Vpeak) [20,21,22]. Data was collected during what was considered to be good weather and satellite conditions for GPS (number of satellites = 20 ± 1.5).

### 2.3. Statistical Analyses

Descriptive results are presented as means ± standard deviation (SD). The distribution was examined using the Shapiro–Wilk normality test and the homogeneity of variance was verified using Levene’s test. A paired t-test for independent samples was used to compare physical demands between floaters and regular players during LSG and SSG. Significant level was stablished at *p* ≤ 0.05. We opted to use effect sizes (ES) with the uncertainty of the estimates shown as 90% confidence intervals (CI) to examine the magnitude of the differences (standardized differences and changes in means) in the physical demands among regular and floater players during different SG formats and between SG formats and match-play. ESs were classified as trivial (<0.2), small (0.2–0.6), moderate (0.6–1.2), and large (>1.2) [23]. These changes were then qualified via probabilistic terms and assigned using the following scale: 25–75%, possibly; 75–95%, likely; 95–99.5%, very likely; and >99.5%, most likely. Inference was classified as unclear if the probability was >5% to overlap the thresholds for the smallest worthwhile positive and negative effects [23].

## 3. Results

Table 3 shows the descriptive results of the physical demands encountered by floaters and regular players in the 8vs8 + 1 LSG, the 4vs4 + 2 SSG and match-play.

**Figure 1:**
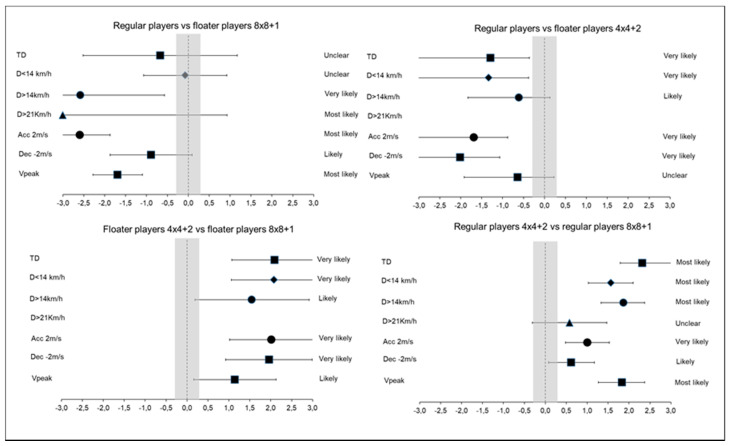
Forest plot showing the effect sizes (with associated confidence intervals) for the physical demand comparisons between regular players and floater players in SSGs. The shaded area, spanning 0.2 to −0.2, represents nonmeaningful effect size. TD: total distance D: Distance; Acc: Acceleration; Dec: Deceleration; Vpeak: Peak velocity.

Table 4 and Figure 1 presents the comparisons among floaters and regular players in the physical demands during the 8vs8 + 1 LSG and the 4vs4 + 2 SSG. However, unclear differences were reported in the TD/min y D < 14.4/min variables in this SG format. Likely–very likely differences were found in the TD/min (ES: −1.29), D < 14.4/min (ES: −1.33), D > 14.4/min (ES: −0.61), Acc > 2/min (ES: −1.68) and Dec > 2/min (ES: −2.01) in regular players in comparison to floaters in the 4vs4 + 2 SSG (Figure 1), but unclear differences were reported in D > 21/min and Vpeak.

**Figure 2:**
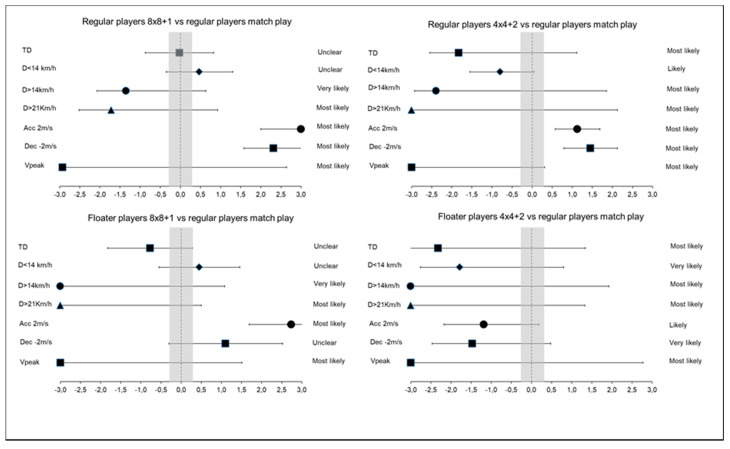
Forest plot showing the effect sizes (with associated confidence intervals) for the physical demand comparisons between regular players and floater players in SSGs and match-play. The shaded area, spanning 0.2 to −0.2, represents nonmeaningful effect size. TD: total distance D: Distance; Acc: Acceleration; Dec: Deceleration; Vpeak: Peak velocity.

Table 5 shows the comparisons in the physical demands encountered by floaters and regular players between the 8vs8 + 1 LSG and the 4vs4 + 2 SSG and the match-play. During match-play, players showed most likely–probably differences in D > 14.4/min (ES: 4.72), D > 21/min (ES: 24.24) and Vpeak (ES: 7.44) in comparison to floaters in the 8vs8 + 1 LSG, but unclear differences were reported in TD/min, D < 14.4/min and Dec > 2/min (Figure 2).

## 4. Discussion

The aims of this study were (1) to analyze the physical demands in the RTP process of high-level soccer players in the role of floater in different SG formats (i.e., 4vs4 + 2 and 8vs8 + 1); and (2) to analyze the differences in physical demands encountered by regular and floater players among the SG formats and official matches. The main results showed lower physical demands for floaters compared to regular players in the SG formats analyzed, higher physical demands for floaters in the 8vs8 + 1 LSG format in comparison to 4vs4 + 2 SSG format, lower distance at high-intensity (D > 14.4/min), high-speed running (D > 21/min), and Vpeak for floaters and regular players during SGs than during match-play, and higher Acc > 2/min and Dec > 2/min for regular players during SGs in comparison to match-play.

Regarding the physical demands encountered by floaters versus regular players, our results are in consonance with those recently found by Lacome et al. (2018) and Rábano-Muñoz et al. (2019). During the 4vs4 + 2 SSG format, the floaters showed lower physical demands than the regular players in terms of TD/min, D < 14.4/min, D > 14.4/min, D > 21/min, Acc > 2/min, and Dec > 2/min. These results could be explained due to the non-participation of floaters in the defensive phase and attack–defence transitions, since their actions are only focused on supporting offensive players and finding free spaces in the playing area. In addition, the non-participation of the floaters may lead them to self-regulation of their efforts, which could explain the decrease in the physical demands that are imposed on the floaters in the 4vs4 + 2 SSG format. These results coincide with in a very similar SSG format (4 vs. 4 + 2 in 30 × 40 m.). Furthermore, most of the physical demands analyzed in this study were lower for floaters than for regular players during the 8vs8 + 1 LSG format, except for the TD/min and D < 14.4/min. However, Lacome, et al. (2018) found that the TD/min and distance at high-intensity (14 km·h^−1^) were lower in floaters compared to regular players, both in LSG and SSG formats. It seems that the presence of a unique floater in the format of 8vs8 + 1 LSG format may explain these differences [9,11]. Since there is a lower numerical superiority, a unique floater, this player usually offers tactical solutions and support to the offensive player maintaining a rhythm during the development the game and provoking similar TD/min and D > 14.4/min. It is very similar to that of normal field players. On the other side, the reduced playing area of the SG selected for this investigation may have influenced both results to achieve higher Vpeak [24], since both floaters and regular players showed similar values. Furthermore, the results obtained in our study contrast with those found by Hill-Haas et al. (2010), who found that the floaters increased their physical demands in comparison to regular players during SSG. The floaters covered greater TD in an SSG format and higher sprints in a MSG format [10]. However, these results should be interpreted with caution due to the low sample size [10] as well as the use of GPS devices of 1 Hz [13]. Finally, considering no study reported higher physical demands for floaters than for regular players [9,11], it would be interesting to use the floater role to modulate the physical demands of the players [11].

Knowing the physical demands on floaters in the different SG formats maintaining the same playing area would be interesting for coaches in order to locate the training tasks within the weekly microcycle [25]. In this sense, our study showed that floaters in the 8vs8 + 1 LSG format had greater physical demands than floaters in the 4vs4 + 2 LSG format in all analyzed variables. Although the floaters in the 8vs8 + 1 LSG play in a smaller area per player than in the 4vs4 + 2 SSG, it seems that the physical demands are similar. In this line, we can suggest that increasing the number of floaters could reduce the physical demands because the numerical superiority causes less mobility to create that game advantage. In this respect [26] found more successful passes in the 5vs5 SG format in comparison to 7vs7. Likewise, our results on the 8vs8 + 1 LSG format may produce a greater number of wrong passes, more turnovers, more technical errors, greater transitions, and a greater exchange of possessions than in the 4vs4 + 2 SSG format, which could explain its increase in the physical demands [27]. The 4vs4 + 2 SSG allows players fewer mistakes and, consequently, fewer transitions because the game is better structured and positional. This fact allows floater players to get the objective of the SG requiring lower physical demands [28].

Quantifying the physical demands of soccer players during SGs and matches provides specific information for the design of training programmes [25]. Our results showed lower physical demands encountered by floaters and regular players during both SSG and LSG in the D > 14.4/m, DT > 21/m and Vpeak in comparison to match-play. The reduced playing area may be the main limiting factor to explain these results, since these SG formats do not allow players to reach high-speeds [6]. Therefore, large playing areas (i.e., higher than 200 m^2^ per player) and higher numbers of players per team (i.e., higher than 8vs8) should be prescribed along the competitive microcycle to overstimulate the high speeds [13,29,30]. On the contrary, higher Acc > 2/min and Dec > 2/min were registered for floaters and regular players during the 8vs8 + 1 LSG format in comparison to match-play. The reduced area per player may explain these differences as previous studies show higher accelerations and decelerations [10,17]. This could be an interesting strategy to overload the neuromuscular system of the players through a higher incidence of accelerations, decelerations and changes of direction at high intensity during central sessions of the microcycle.

Considering the physical demands encountered by players in the different SSG, LSG, and matches analyzed in our study, it seems that the players in the RTP process could carry out the following phases: (1) floater in the 4vs4 + 2 SSG; (2) regular player in 4vs4 + 2 SSG; (3) floater in the 8vs8 + 1 LSG; and (4) regular player in 8vs8 + 1 LSG before returning to competition. Providing a progressive reintroduction to the training process is a crucial issue for coaches. From a practical point of view, the results provide a specific strategy to optimise the RTP in soccer players who have suffered muscular injuries, especially those injuries at posterior chain. This proposal allows players to minimise the exposure to external loads in terms of high-intensity actions, while progressively increasing the physical demands during the training process. Since no TD > 21/min was recorded during both SG (i.e., SSG and LSG), it would be advisable for players to perform analytical tasks in a progressive manner that stimulate physical demands similar to matches at high speeds [21]. The use of soccer tasks which demand displacements of high speed (i.e., cruising and sprinting activities) seems to be necessary to exert a protective effect against on soccer players against suffering injuries [13]. It could be that the implementation of these analytical tasks to achieve high-intensity actions allows players to progressively gain confidence and feel more mentally prepared to compete. To perform high-speed running reduces the variability in the individual responses [27], which implies to control the players’ training dose during the RTP since the specific training based on SG could produce an opposite effect, increasing the variability of the responses [10]. This fact could have an impact on performing efforts for which the players may not yet be prepared, so this combination could be interesting for the last phase of the RTP, before including the injured player in full trainings and match-play.

It has been demonstrated that the analysis of short-term and high-intensity actions in soccer, such as accelerations and decelerations, is crucial to optimise performance [25,28]. Adopting the results obtained in this study in terms of Acc > 2/min and Dec > 2/min, the progression to lead the RTP should be (1) floater in the 4vs4 + 2 SSG; (2) floater in the 8vs8 + 1 LSG; (3) regular player in the 4vs4 + 2 SSG where the player increases the Acc > 2/min and Dec > 2/min performed during match-play; and (4) floater in the 8vs8 + 1 LSG before starting full trainings and returning to competition. This RTP ensures a progressive reintroduction in terms of acceleration and deceleration variables. From a practical point of view, these results allow coaches to individualise the training load and schedule a progression in the RTP phases, especially in players who have suffered joint injuries.

The main limitation of this study was that only the external loads were quantified; however, it could be interesting to know the internal loads in order to better understand the acute effects of the different SG formats. One limitation was that only two SG formats (i.e., 4vs4 + 2 and 8vs8 + 1) played in the same playing area have been used, and knowing the physical demands encountered by soccer players in larger areas and different orientations could provide useful information for coaches, specifically during the RTP process. Ultimately, none of the players involved in this study was in the return-to-play process, leaving therefore an outstanding starting point for future works.

## 5. Conclusions

The results of the present study demonstrate that the use of floaters decreases the physical demands in comparison to those demands encountered by regular players during the SGs formats (i.e., 4vs4 + 2 SSG and 8vs8 + 1 LSG). Regular players in 8vs8 + 1 LSG increase the physical demands in comparison to regular players in 4vs4 + 2 SSG. In addition, distance at high-intensity (D > 14.4/min), high-speed running (D > 21/min) and Vpeak for floaters and regular players during SGs is lower than during match-play, and Acc > 2/min and Dec > 2/min for regular players is higher during SGs in comparison to match-play. Therefore, the use of floaters during the last phase of the RTP seems to be an adequate strategy for progressive reintroduction into specific training.

## Figures and Tables

**Table 1:** Characteristics of the participants.

Group	*Age (Years)*	*Height (cm)*	*Body Mass (kg)*	*Sitting Height (cm)*	*Leg Length (cm)*	*APHV (Years)*
Floater players (*n* = 3)	15.9 ± 0.1	172.4 ± 4.1	68.8 ± 5.3	90.6 ± 1.4	81.8 ± 2.9	14.0 ± 0.3
Regular players (*n* = 23)	15.7 ± 0.3	176.6 ± 5.9	67.9 ± 5.9	91.9 ± 3.2	84.7 ± 3.2	13.7 ± 0.5
Total players (*n* = 26)	15.7 ± 0.3	176.1 ± 5.8	68.0 ± 5.6	91.7 ± 3	84.4 ± 3.2	13.8 ± 0.4

APHV. Age at peak height velocity.

**Table 2:** Characteristics of the soccer sided games.

	Duration (min)	Players (*n*)	Dimensions (m)	Playing Area (m^2^)	Area per Player (m^2^)
LSG 8vs8 + 1	6	17	30 × 25	750	44.1
SSG 4vs4 + 2	6	10	30 × 25	750	75.0
Match-play 11vs11	80	22	100 × 60	6000	272.7

LSG = large-sided game; SSG: small-sided game.

**Table 3:** Descriptive results in the physical demands encountered by floaters and regular soccer players during the 8vs8 + 1 LSG, the 4vs4 + 2 SSG and match-play.

	8vs8 + 1 LSG		4vs4 + 2 SSG		Match-Play
	Floater (*n* = 1)	Regulars (*n* = 9)	Floaters (*n* = 2)	Regulars (*n* = 8)	Regulars (*n* = 7)
TD/min	91.31 ± 5.20	101.66 ± 8.75	49.14 ± 15.14	74.10 ± 12.68	102.65 ± 14.66
D < 14.4/min	88.98 ± 4.58	90.15 ± 8.92	48.53 ± 15.46	73.19 ± 11.97	84.34 ± 13.08
D > 14.4/min	2.24 ± 0.51	11.21 ± 4.21	0.59 ± 0.66	2.92 ± 3.01	18.31 ± 5.52
D > 21.0/min	0.00 ± 0.00	0.78 ± 1.23	0.00 ± 0.00	0.05 ± 0.15	4.24 ± 2.26
Acc > 2/min	2.58 ± 0.12	3.80 ± 0.65	1.33 ± 0.53	2.89 ± 0.85	1.93 ± 0.23
Dec > 2/min	2.42 ± 0.35	3.48 ± 0.88	0.81 ± 0.57	2.88 ± 0.80	1.81 ± 0.41
Vpeak	18.96 ± 0.18	22.08 ± 2.49	16.01 ± 3.03	17.85 ± 2.08	29.32 ± 2.14

LSG: large-sided game; SSG: small-sided game; TD: total distance D: Distance; Acc: Acceleration; Dec: Deceleration; Vpeak: Peak velocity.

**Table 4:** Comparisons among floater and regular players in the physical demands during the 8vs8 + 1 LSG and the 4vs4 + 2 SSG.

	8vs8 + 1: Regular vs. Floater Players	4vs4 + 2 Regular vs. Floater Players	Floaters vs. Floater 4vs4 + 2 and 8vs8 + 1	Regular vs. Regular 4vs4 + 2 and 8vs8 + 1
TD/min	−9.9 (−32.6; 20.3) *p* = 0.127	−36.3 (−54.1; −11.7) *p* = 0.000	96.2 (41.2; 172.6) *p* = 0.013	38.6 (28.7; 49.3) *p* = 0.000
D < 14.4/min	−0.9 (−13.0; 12.9) *p* = 0.860	−36.3 (−53.8; −12.3) *p* = 0.000	93.2 (40.1; 166.5) *p* = 0.013	24.1 (15.3; 33.5) *p* = 0.000
D > 14.4/min	−78.8 (−93.7; −29.2) *p* = 0.000	−53.4 (−81.6; 17.7) *p* = 0.003	209.5 (14.9; 733.9) *p* = 0.019	581.2 (298.1; 1065.9) *p* = 0.000
D > 21.0/min	−100(−100; −100) *p* = 0.395	-	-	61.7 (−22.4; 237.0) *p* = 0.031
Acc > 2/min	−31.1 (−38.0; −23.7) *p* = 0.020	−54.5 (−68.7; −33.6) *p* = 0.000	108.6 (44.9; 200.3) *p* = 0.019	37.9 (16.7; 63.1) *p* = 0.001
Dec > 2/min	−28.4 (−50.6; 3.6) *p* = 0.118	−77.1 (−88.6; −54.3) *p* = 0.000	282.4 (88.2; 676.6) *p* = 0.011	22.1 (2.6; 45.3) *p* = 0.038
Vpeak	−13.1 (−17.3; −8.8) *p* = 0.103	−11.1 (−24.2; 4.3) *p* = 0.097	20.2 (2.6; 40.8) *p* = 0.063	23.8 (16.1; 31.9) *p* = 0.00

LSG: large-sided game; SSG: small-sided game; TD: total distance D: Distance; Acc: Acceleration; Dec: Deceleration; Vpeak: Peak velocity.

**Table 5:** Comparisons in the physical demands encountered by floater and regular players between the 8vs8 + 1 LSG and the 4vs4 + 2 SSG and the match-play.

	8vs8 + 1 Regular vs. Regular Match	4vs4 + 2 Regular vs. Regular Match	Floater 8vs8 + 1 vs. Regular Match	Floater 4vs4 + 2 vs. Regular Match
TD/min	−10.2 (−22.5; 4.1) *p* = 0.840	−54.2 (−67.1; 36.4) *p* = 0.337	−0.3 (−11.8; 12.7) *p* = 0.000	−28.1 (−36.8; −18.2) *p* = 0.000
D < 14.4/min	6.6 (−7.5; 23.0) *p* = 0.226	−44.8 (−60.2; 23.5) *p* = 0.651	7.6 (−5.3; 22.3) *p* = 0.047	−13.3 (−24.1; −0.9) *p* = 0.001
D > 14.4/min	−87.4 (−93.0; −77.2) *p* = 0.006	−95.9 (−98.4; −89.4) *p* = 0.000	−40.6 (−55.0; −21.5) *p* = 0.000	−91.3 (−95.0; −84.9) *p* = 0.000
D > 21.0/min	−100(−100; −100) *p* = 0.000	−100(−100; −100) *p* = 0.003	−78.2 (−89.2; −56.0) *p* = 0.003	−86.5 (−93.2; −73.1) *p* = 0.003
Acc > 2/min	34.7 (20.2; 51.1) *p* = 0.000	−35.4 (−55.4; −6.4) *p* = 0.008	95.7 (75.0; 118.7) *p* = 0.007	41.9 (19.6; 68.3) *p* = 0.040
Dec > 2/min	36.0 (−8.6; 102.4) *p* = 0.000	−64.4 (−82.3; −28.4) *p* = 0.102	90.0 (55.4; 132.3) *p* = 0.002	55.6 (27.2; 90.3) *p* = 0.004
Vpeak	−35.3 (−38.8; −31.7) *p* = 0.000	−46.2 (−54.1; −36.8) *p* = 0.000	−25.1 (−30.0; −19.8) *p* = 0.000	−39.5 (−43.3; −35.4) *p* = 0.000

LSG: large-sided game; SSG: small-sided game; TD: total distance D: Distance; Acc: Acceleration; Dec: Deceleration; Vpeak: Peak velocity.
